# Agronomic Performance of Microbial Inoculation Strategies in Soybean, Maize and Wheat Across Contrasting Environments

**DOI:** 10.3390/plants15193025

**Published:** 2026-10-02

**Authors:** André Sarabia Zamarian, Ana Claudia Botelho, Ricardo Robson Trautmann, Tauane Santos Brito, Andre Gustavo Battistus

**Affiliations:** 1 Tecnomyl S.A., Av. Aviadores del Chaco n° 3301, Asunción 1110, Paraguay; tauane.brito@tecnomyl.com (T.S.B.); andre.battistus@tecnomyl.com (A.G.B.); 2 Tecnomyl Brasil, St. Professor Joao Argemiro Loyola, n° 319, Seminario, Curitiba 80240-530, Paraná, Brazil; ana.botelho@tecnomyl.com; 3 Agrofértil, Av. San Blas/Pablo Neruda-Km. 6,5, Ciudad del Este 7000, Alto Paraná, Paraguay; ricardo.trautmann@agrofertil.com.py

**Keywords:** growth-promoting bacteria, rhizobacteria, nitrogen fertilization, plant–bacterial association

## Abstract

This study evaluated the effects of co-inoculation with *Azospirillum brasilense* (strains AbV5 and AbV6) and *Bradyrhizobium* spp. (*B. japonicum* SEMIA 5079 and *B. diazoefficiens* SEMIA 5080) on soybean, as well as the response of maize and wheat to *A. brasilense* inoculation at different nitrogen fertilization levels. Twelve field trials were conducted across four contrasting edaphoclimatic regions during the 2023/2024 growing season using randomized block designs with four replicates. The treatments represented predefined inoculation and nitrogen-management strategies rather than a full factorial design; therefore, the study was intended to compare agronomic management strategies rather than estimate statistical interactions between inoculation and nitrogen fertilization. Co-inoculation in soybean increased the pod number, thousand-grain weight, and grain yield. In maize and wheat, inoculation associated with nitrogen fertilization resulted in significant increases in biomass accumulation and grain yield compared with the uninoculated control. These results indicate that microbial inoculation can improve selected agronomic traits under the field conditions evaluated. In cereals, the association of *A. brasilense* with different nitrogen fertilization levels improved selected agronomic responses compared with the uninoculated control under the field conditions evaluated, although the magnitude of these responses varied among nitrogen-management strategies and production environments.

## 1. Introduction

Soybean, maize, and wheat are major grain crops in Brazilian agricultural systems and play important roles in food, feed, and agroindustrial production. The increasing demand for higher productivity in these crops has intensified the search for sustainable agronomic strategies, including the use of plant growth-promoting bacteria (PGPB). Among these, *Azospirillum brasilense* has been widely studied due to its ability to stimulate root development and influence plant growth through phytohormone production, potentially enhancing nutrient uptake and plant performance under field conditions [1,2,3].

In cereal crops, inoculation with *A. brasilense* has been associated with improvements in morphophysiological traits, nutrient use, and yield components [4,5,6,7]. In soybean, co-inoculation with *Bradyrhizobium* spp., such as *B. japonicum* and *B. diazoefficiens*, has been adopted as a strategy to enhance biological nitrogen fixation and improve plant development. This association is often attributed to the stimulation of root growth by *A. brasilense*, which may favor nodulation and nutrient acquisition [3,8].

More broadly, plant-associated microorganisms and their interactions with host plants represent an important component of strategies aimed at improving crop performance and promoting more sustainable agricultural systems [9,10]. However, the complexity of plant–microbe interactions and the limited persistence and consistency of introduced microorganisms under variable environmental conditions remain important challenges for the field application of microbial technologies [9,10].

Despite these advances, most studies have been conducted under specific crop conditions or single environments, and there is still limited information regarding the agronomic performance of these inoculation strategies across multiple crops and contrasting edaphoclimatic conditions. In addition, the agronomic performance of microbial inoculation associated with different nitrogen fertilization levels in cereal systems remains insufficiently explored under field conditions.

We hypothesized that crop-specific microbial inoculation strategies would improve selected agronomic traits compared with the uninoculated control under the evaluated field conditions, with co-inoculation strategies in soybean and *A. brasilense*-based nitrogen-management strategies in cereals affecting biomass accumulation, yield components, and grain yield.

Therefore, this study aimed to evaluate the agronomic responses to inoculation with *Azospirillum brasilense* (strains AbV5 and AbV6), alone or when co-inoculated with *Bradyrhizobium* spp. (*B. japonicum* SEMIA 5079 and *B. diazoefficiens* SEMIA 5080), in soybean, maize, and wheat cultivated under different environmental conditions, as well as to assess the performance of *A. brasilense*-based management strategies associated with different nitrogen fertilization levels in cereal crops.

## 2. Materials and Methods

### 2.1. Characterization of Experimental Areas

During the 2023/2024 harvest, twelve trials were conducted on maize, wheat and soybean crops in four edaphoclimatic regions detailed in Table 1.

In the municipality of Artur Nogueira, São Paulo, the tests were implemented at latitude 22°31′52.53″ S and longitude 47°07′5.11″ W, with an altitude of 596 m. According to Köppen [11], the climate of the region is classified as tropical with a dry winter (Aw), with an average temperature of 21.7 °C and an average precipitation of 1484 mm. The soil is classified as Acriferric Red Latosol with a clayey texture [12] and a pH of 4.20.

In the municipality of Bandeirantes, Paraná, the tests were implemented at latitude 23°6′53.18“ S and longitude 50°21′22.74″ W, with an altitude of 449 m. According to Köppen [11], the climate of the region is classified as tropical (Aw), with an average temperature of 22.2 °C and an average precipitation of 1339 mm. The soil is classified as Eutroferric Red Latosol, with a very clayey texture [12] and a pH of 5.73.

In the municipality of Ouro, Santa Catarina, the tests were implemented at latitude 27°18′14.11″ S and longitude 51°37′56.29″ W, with an altitude of 526 m. According to Köppen [11], the climate of the region is classified as humid subtropical (Cfa), with an average temperature of 22 °C and an average precipitation of 1210 mm. The soil is classified as moderate Eutrophic Cambisol Tb A, with a clayey texture, and moderate Eutrophic Litholics A, with a medium texture, both stony and in strongly wavy relief [12], with a pH of 4.71.

In the municipality of Ponta Grossa, Paraná, the tests were implemented at latitude 25°9′53.85″ S and longitude 50°11′23.79″ W, with an altitude of 799 m. According to Köppen [11], the climate of the region is classified as temperate humid (Cfb), with an average temperature of 18.9° C and an average precipitation of 1371 mm. The soil is classified as a typical Tb Eutrophic Haplic Cambisol, with a clayey texture [12] and a pH of 5.35.

The experimental sites differed substantially in their edaphoclimatic conditions, including climate classification, temperature, rainfall, altitude, soil type, texture, and soil pH. Soil acidity may influence the establishment and activity of *A. brasilense*; however, the agronomic responses observed across locations cannot be attributed exclusively to pH. Plant–bacterial interactions under field conditions result from the combined influence of soil, climatic, crop, and management factors. Although background populations of culturable diazotrophic bacteria were estimated at sowing, bacterial survival, root colonization, and population dynamics following inoculation were not quantified. Consequently, differences in agronomic responses among environments cannot be directly attributed to differences in bacterial establishment, persistence, or activity. These responses should therefore be interpreted as the overall agronomic outcome of the inoculation strategies under contrasting field conditions.

Background populations of culturable diazotrophic bacteria were estimated at sowing using the most probable number (MPN) method in a semi-solid NFb medium. The estimated populations varied among crops and experimental sites (Table 2). This assessment represents the background culturable diazotrophic bacterial population and was not specific to soybean-nodulating rhizobia or native *Bradyrhizobium* spp.

### 2.2. Experimental Design and Treatments

The trials were conducted in a randomized block design. For soybean, six treatments with four replicates were used, whereas maize and wheat comprised eight treatments with four replicates (Table 3). Experimental plot dimensions and useful areas were established according to each crop and experimental protocol. All experiments were conducted under rainfed field conditions, without supplemental irrigation. Phytosanitary management was performed as needed according to each crop and experimental location.

The bacterial treatments were applied using ready-to-use inoculant formulations containing the *Azospirillum brasilense* strains AbV5 and AbV6 and *Bradyrhizobium japonicum* SEMIA 5079 and *Bradyrhizobium diazoefficiens* SEMIA 5080 at concentrations of 2.0 × 10^9^ and 2.0 × 10^10^ CFU mL^−1^, respectively. These strains were obtained from the Diazotrophic and Plant Growth-Promoting Bacteria Culture Collection of EMBRAPA Soybean, Londrina, Paraná, Brazil. The inoculants were applied manually as seed treatments at the doses specified for each treatment, without the addition of adherence promoters or seed protectants, and were stored according to the manufacturers’ recommendations until application.

For maize and wheat, the treatments represented predefined management strategies involving microbial inoculation and different nitrogen fertilization levels and were not structured as a full factorial experiment. The treatment designated as “Commercial product” and the treatment designated as “*Azospirillum brasilense*” contained the same bacterial strains (AbV5 and AbV6) and were applied at the same nominal dose but differed in the formulation medium used to deliver the microorganisms. The commercial product was included as a positive control, representing an established *A. brasilense*-based inoculant available on the market, whereas the *A. brasilense* treatment corresponded to the inoculant evaluated in the experimental treatments. Thus, although both treatments had the same bacterial composition, they represented distinct inoculant formulations. Detailed information regarding the manufacturing process and formulation of the commercial product was not available to the authors. Comparisons among treatments were therefore intended to evaluate the agronomic performance of the individual management strategies rather than factorial interactions between inoculation and nitrogen fertilization.

For yield assessment, only the central portion of each plot was harvested to minimize potential edge effects. The harvested area was 7.5 m^2^ for soybean and 3.0 m^2^ for maize and wheat according to the crop-specific plot configuration and planting arrangement.

#### 2.2.1. Soybean

For the soybean tests, the cultivar HO Pirapó IPRO was used. The base fertilization dose used in Ponta Grossa–PR and Bandeirantes–PR was 240 kg ha^−1^ of the 00-20-20 NPK formulation, and, in Ouro–SC and Artur Nogueira–SP, it was 300 kg ha^−1^ of the same formulation. The sowing protocol followed the spacing of 50 cm between rows, resulting in 300,000 plants ha^−1^. Treatments and their respective microbial and fertilizer components are presented in Table 3.

In soybean, five whole plants were collected from the central area of each experimental plot, outside the area designated for grain yield assessment, at the V4 (BBCH 14), flowering (BBCH 60), and R3 (beginning pod filling; BBCH 70) stages. Shoot and root fresh masses were determined, after which the plant material was dried in an oven at 65 °C until a constant weight was achieved to determine shoot and root dry masses. Five plants per plot were also evaluated for the number of nodules at 35 days after emergence. At harvest, plant population, the number of pods per plant, the number of grains per pod, thousand-seed weight (TSW), and grain yield were determined. Grain yield was evaluated from a 7.5 m^2^ area per plot; grains were cleaned and weighed, moisture content was adjusted to 13%, and yield was expressed as kg ha^−1^. Leaf and grain nitrogen, phosphorus, and potassium contents were also determined.

The treatments were defined as predefined management strategies commonly adopted under commercial production systems rather than as a factorial arrangement, aiming to evaluate the agronomic performance of inoculation practices under field conditions.

The 200 kg N ha^−1^ rate was included as a high-mineral-N reference treatment and was evaluated separately from the microbial inoculation treatments; this rate was not intended to represent a recommended N fertilization practice for soybean.

#### 2.2.2. Maize and Wheat

The maize hybrid used was Ferocious Vip 3. The base fertilization dose used in Ponta Grossa–PR and Ouro–SC was 300 kg ha^−1^ of the formulated 00-20-20 NPK; in Bandeirantes–PR, it was 240 kg ha^−1^ of the same formulation, and in Artur Nogueira–SP, it was 300 kg ha^−1^ of the formulated 00-21-21 NPK. The sowing protocol followed the spacing of 50 cm between rows, resulting in 70,000 plants ha^−1^.

In maize, five whole plants were collected from the central area of each experimental plot, outside the area designated for grain yield assessment, at the V4 (BBCH 14), flowering (BBCH 63), and R3 (milky grain; BBCH 73) stages. Shoots and roots were separated and dried in an oven at 65 °C until a constant weight was achieved to determine shoot and root dry mass. At harvest, grain yield was determined from a 3.0 m^2^ area within each plot. Grains were cleaned and weighed, moisture content was adjusted to 13%, and grain yield was expressed as kg ha^−1^. Thousand-seed weight (TSW), the number of grain rows per ear, and the number of grains per row were also determined. Leaf and grain nutritional contents were also evaluated.

The wheat cultivar used was the TBIO Ponteiro. The base fertilization dose used was 300 kg ha^−1^ of the 10-20-10 NPK formulation at the four sites. The sowing protocol followed the spacing of 17 cm between rows with an average of 70 plants m^−1^. Table 4 describes the treatments used in both experiments.

In wheat, shoot and root dry masses were evaluated at tillering (BBCH 29), flowering (BBCH 61), and maturation (BBCH 83) using 10 whole plants per experimental plot. Plants were collected from the central area of each plot, outside the area designated for grain yield assessment. Plant material was separated into shoots and roots and dried in an oven at 65 °C until a constant weight was achieved. At harvest, the number of plants per square meter, the number of ears per square meter, the number of spikelets per ear, the number of grains per ear, and grain yield were determined. Grain yield was evaluated from a 3.0 m^2^ area per plot; grains were cleaned and weighed, moisture content was adjusted to 13%, and yield was expressed as kg ha^−1^.

### 2.3. Weather

Weather data were obtained from a meteorological station located at or near each experimental site. Data were recorded from the beginning of the sowing month until crop harvest. Monthly mean air temperature and accumulated precipitation during the experimental period are presented in Figure 1.

### 2.4. Statistical Analysis

The data were subjected to tests of normality and homogeneity of variances prior to analysis. A joint analysis across locations was performed, considering each location as a distinct production environment. Analysis of variance (ANOVA) was conducted separately for each crop. For variables evaluated at multiple phenological stages, the plants sampled within each plot were treated as subsamples and used to obtain a plot-level mean. Each phenological stage was analyzed independently, with the plot considered the experimental unit, and no repeated-measures analysis was performed. When the ANOVA was significant at a 5% probability, treatment means were compared using Dunnett’s test at a 95% confidence level, considering the control treatment as the reference. In the joint analysis, locations were considered random effects and treatments were considered fixed effects, allowing the overall treatment response to be evaluated across the contrasting production environments. The statistical analyses were performed using R software, version 4.5.1.

## 3. Results

### 3.1. Soybean

There was an increase in the number of pods per plant in the treatments with nitrogen fertilization and a standard dose of *Bradyrhizobium* (*B. japonicum* + *B. diazoefficiens*) and *Bradyrhizobium* + *Azospirillum* (2:1) in relation to the uninoculated control (Table 5). The thousand-seed weight (TSW) was statistically superior to that of the control for the standard-dose treatments of *Bradyrhizobium* (*B. japonicum* + *B. diazoefficiens*), *Bradyrhizobium* + *Azospirillum* (1:1), and *Bradyrhizobium* + *Azospirillum* (2:1) (Table 5).

There was a significant increase in productivity in relation to the control for the nitrogen fertilization, *Bradyrhizobium*, *Azospirillum*, and *Bradyrhizobium* + *Azospirillum* (2:1) treatments (Table 5).

The variables fresh mass of aerial parts and roots, dry mass of aerial parts and roots, number of nodules, and leaf and grain nitrogen, phosphorus, and potassium contents did not show significant increases with the application of treatments containing *Bradyrhizobium* (*B. japonicum* + *B. diazoefficiens*), either alone or when co-inoculated with *Azospirillum brasilense*, in the phenological stages evaluated.

### 3.2. Maize

At the V4 and flowering stages, shoot dry mass in maize was significantly higher than that of the control in all treatments except the treatment receiving 50% nitrogen fertilization without *Azospirillum brasilense* inoculation (Table 6). At the R3 stage, only the commercial product treatment resulted in significantly greater shoot dry mass than the control (Table 6).

The accumulation of root dry mass was statistically higher than that in the control at V4 when 100% nitrogen fertilization was associated with seed inoculation with *A. brasilense*. During flowering, significant increases were observed in the treatments combining *A. brasilense* with 75% or 100% nitrogen fertilization. At the R3 stage, significant increases in root dry mass compared with the control were observed for 100% nitrogen fertilization, *A. brasilense* inoculation, and *A. brasilense* inoculation with 50%, 75%, or 100% nitrogen fertilization (Table 6).

For the production components, the number of grains per row was statistically superior to that of the control in the treatments with 100% nitrogen fertilization and *A. brasilense* + 100% N. Thousand-seed weight was statistically superior to that of the control in the treatment with *A. brasilense* + 75% N. Productivity was significantly higher than that of the control in the treatments with 100% nitrogen fertilization and *A. brasilense* plus 50%, 75%, or 100% nitrogen fertilization (Table 7).

No significant differences were observed for the number of grain rows per ear or for the nutritional content of leaves and grains.

### 3.3. Wheat

At tillering, there was an increase in the dry mass of the aerial part of plants that received inoculation with *Azospirillum brasilense*, alone and in association with 75% of the nitrogen dose, compared to the control. During flowering, there was an increase in the dry mass of the aerial part in the treatment with 100% nitrogen fertilization and in treatments containing *A. brasilense*, alone or in combination with 50%, 75%, or 100% nitrogen fertilization. At maturation, this increase was observed in the treatments with 100% nitrogen fertilization and in those associating *A. brasilense* with 75% or 100% nitrogen fertilization (Table 8).

The accumulation of root dry mass was statistically higher than that in the control in plants inoculated with *A. brasilense* along with 75% or 100% nitrogen fertilization at tillering. During flowering, significant increases in root dry mass compared with the control were observed in the 100% nitrogen fertilization and commercial product treatments, as well as in all treatments containing *A. brasilense*. At maturation, significant increases compared with the control were observed for 100% nitrogen fertilization and for *A. brasilense* plus 75% or 100% nitrogen fertilization (Table 8).

Inoculation with *A. brasilense* along with 50%, 75%, or 100% nitrogen fertilization resulted in statistically significant increases in the number of ears per square meter, the number of spikelets per ear, and the number of grains per ear in relation to the control. Productivity was significantly higher than that of the control in all evaluated treatments (Table 9).

## 4. Discussion

The effects of *Azospirillum brasilense* on crop performance have been associated in previous studies with several plant growth-promoting mechanisms, including phytohormone-related responses, nutrient acquisition, and changes in root development [1,13,14]. The AbV5 and AbV6 strains used in the present study have been previously evaluated as plant growth-promoting inoculants in maize and have been associated with improved fertilizer-derived nitrogen recovery and grain yield [15]. These findings support their use in inoculation strategies aimed at improving nitrogen acquisition and crop performance. In the present study, inoculation with *A. brasilense*, alone or in combination with nitrogen fertilization, was associated with increases in shoot and root biomass and grain yield in maize and wheat. These responses are consistent with previous evidence of beneficial effects of AbV5 and AbV6 on maize performance [15]; however, physiological mechanisms such as phytohormone production, biological nitrogen fixation, and changes in nutrient uptake efficiency were not directly quantified in the present study.

In soybean, the SEMIA 5079 and SEMIA 5080 strains are established *Bradyrhizobium* strains used in soybean inoculation because of their symbiotic relationship with soybean and contribution to biological nitrogen fixation. Co-inoculation of *Bradyrhizobium* with plant growth-promoting rhizobacteria, including *A. brasilense*, has been reported to improve nodulation and plant development, although responses may vary according to the co-inoculant and experimental conditions [3,8,16]. Previous studies have reported positive effects of *Bradyrhizobium–Azospirillum* co-inoculation on nodulation, root biomass, shoot biomass, and other plant-development traits, although increases in grain yield are not consistently observed across studies. In the present study, inoculation with *Bradyrhizobium* increased the number of pods per plant, thousand-seed weight, and grain yield compared with the uninoculated control. Co-inoculation with *Bradyrhizobium* and *A. brasilense*, particularly at the 2:1 ratio, also resulted in significant increases in these production components compared with the control. These results indicate a positive agronomic response to inoculation and co-inoculation under the evaluated field conditions, although the mechanisms underlying these responses were not directly investigated.

In maize, inoculation with *A. brasilense*, particularly when associated with nitrogen fertilization, resulted in significant increases in shoot and root dry mass at different phenological stages compared with the control. Significant increases in grain yield were also observed when *A. brasilense* was associated with 50%, 75%, or 100% nitrogen fertilization. In wheat, similar responses were observed, with increases in shoot and root dry mass and in several yield components in treatments containing *A. brasilense*. These results demonstrate that the response to inoculation occurred at different stages of crop development and was reflected in agronomic performance at harvest.

The effects of microbial inoculation can vary according to environmental conditions, soil properties, crop characteristics, and management practices [10]. The experimental sites used in the present study represented contrasting edaphoclimatic environments, differing in climate classification, temperature, precipitation, altitude, soil type, texture, and soil pH. Therefore, variation in treatment responses among locations may reflect the combined influence of these environmental factors rather than the effect of a single soil or climatic variable. Although periods of water restriction and high temperatures occurred during the experiments [17,18], the present study was not designed to isolate or quantify the specific effects of these stresses on the response to inoculation.

Overall, the results indicate that inoculation strategies involving *A. brasilense*, either alone or in association with nitrogen fertilization or *Bradyrhizobium* spp., can contribute to improvements in biomass accumulation, yield components, and grain yield under different field environments. However, because the physiological and microbiological mechanisms underlying these responses were not directly evaluated, the observed effects should be interpreted primarily as agronomic responses under the conditions of the present study. The variation observed among environments also reinforces the importance of considering local edaphoclimatic conditions and crop management when evaluating the performance of microbial inoculants.

## 5. Conclusions

The results indicate that inoculation with *Azospirillum brasilense*, alone or in combination with *Bradyrhizobium* spp., can enhance agronomic performance in soybean, maize, and wheat under field conditions. In cereal crops, the association of *A. brasilense* with different nitrogen fertilization levels resulted in significant increases in biomass accumulation, yield components, and grain yield compared with the uninoculated control under the evaluated field conditions.

Positive responses were observed across multiple environments; however, their magnitude varied among crops, inoculation strategies, and production environments, indicating that the benefits of microbial inoculation are not uniform across production systems. This variability highlights the importance of considering local edaphoclimatic conditions and management practices when developing recommendations for microbial inoculants. However, the mechanisms underlying these variable responses remain to be elucidated, particularly because bacterial establishment, persistence, and root colonization were not monitored in the present study. Future research should integrate multi-environment and long-term field evaluations with microbial population dynamics and detailed soil, climatic, and management characterization to identify the conditions under which specific inoculation strategies provide the most consistent agronomic benefits. Such information may contribute to more targeted recommendations for microbial inoculation according to the crop and production environment.

## Figures and Tables

**Figure 1:**
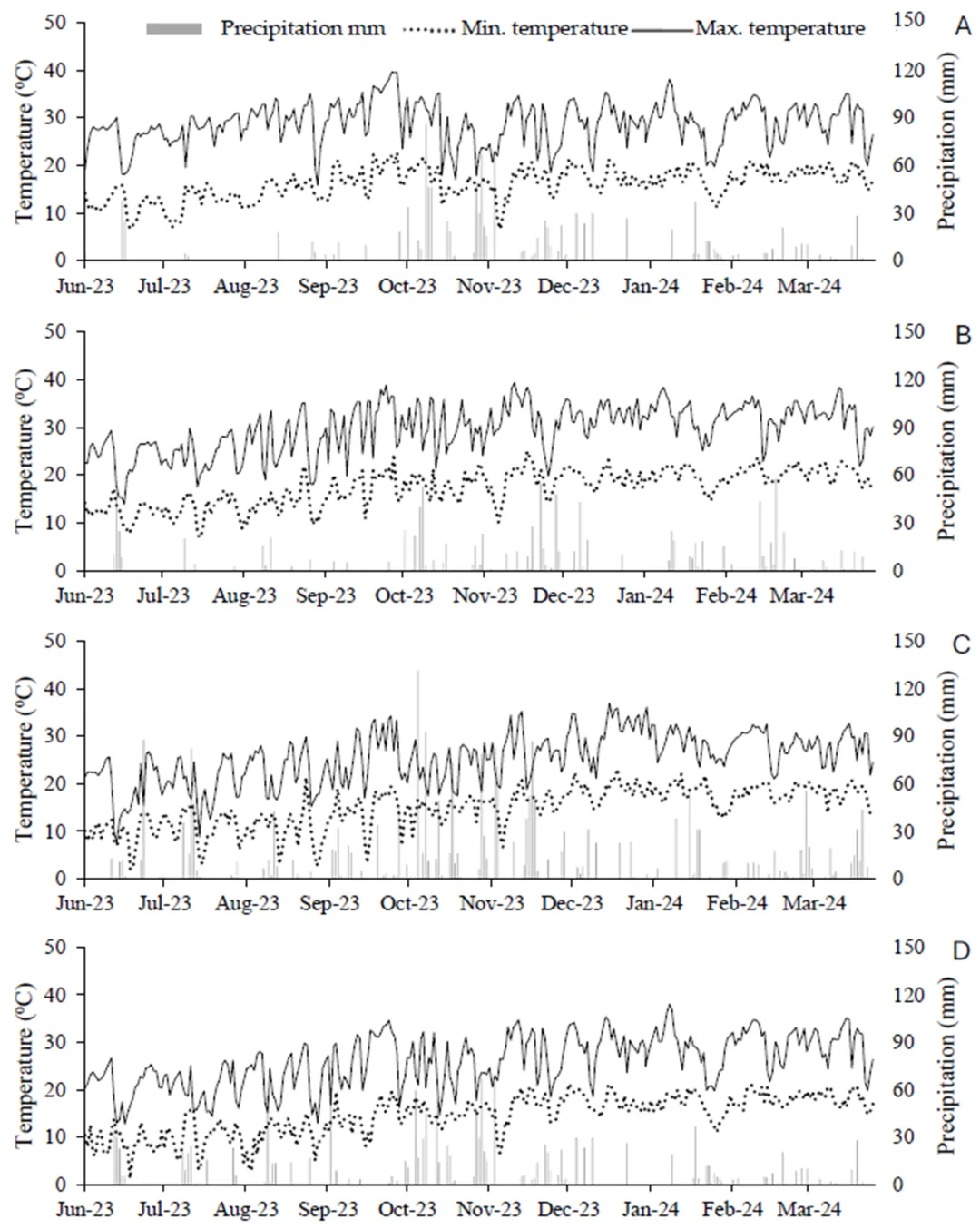
Monthly averages of the average temperature (°C) and accumulated rainfall (mm) in the region of Artur Nogueira–SP (**A**), Bandeirantes–PR (**B**), Ouro–SC (**C**) and Ponta Grossa–PR (**D**) during the experimental period (2023/2024 harvest).

**Table 1:** Sowing and harvesting periods of crops inoculated with *Azospirillum brasilense* under co-inoculation or nitrogen fertilization in the 2023/2024 harvest in different regions.

Crop	Location	Sowing	Harvest
Soybean	Artur Nogueira (SP)	23 October 2023	29 February 2024
	Bandeirantes (PR)	18 October 2023	15 March 2024
	Ouro (SC)	25 October 2023	10 March 2024
	Ponta Grossa (PR)	10 October 2023	14 March 2024
Maize	Artur Nogueira (SP)	23 October 2023	15 March 2024
	Bandeirantes (PR)	3 October 2023	27 March 2024
	Ouro (SC)	25 October 2023	23 March 2024
	Ponta Grossa (PR)	26 September 2023	25 March 2024
Wheat	Artur Nogueira (SP)	22 July 2023	13 October 2023
	Bandeirantes (PR)	24 July 2023	14 October 2023
	Ouro (SC)	24 July 2023	13 October 2023
	Ponta Grossa (PR)	22 July 2023	14 October 2023

**Table 2:** Background populations of culturable diazotrophic bacteria (MPN g^−1^ fresh soil) in soil at sowing in the experimental areas.

Crop	Artur Nogueira (SP)	Bandeirantes (PR)	Ouro (SC)	Ponta Grossa (PR)
Soybean	6.6 × 10^4^	3.0 × 10^4^	1.2 × 10^5^	3.0 × 10^4^
Maize	6.1 × 10^4^	2.2 × 10^5^	1.2 × 10^5^	2.5 × 10^4^
Wheat	6.6 × 10^4^	2.2 × 10^5^	1.7 × 10^4^	5.7 × 10^4^

**Table 3:** Description of the microorganisms and doses used in the trials conducted on soybeans in different regions in the 2023/2024 harvest.

Crop	Treatments	Microorganisms	CFU mL^−1^	Dose
Soybean	Control	-	-	-
	Nitrogen fertilization (N)	-	-	200 kg N ha^−1^
	*Bradyrhizobium*	*Bradyrhizobium japonicum* SEMIA 5079	2.0 × 10^10^	2 mL kg^−1^ seeds
		*Bradyrhizobium diazoefficiens* SEMIA 5080		
	*Azospirillum*	*Azospirillum brasilense* AbV5	2.0 × 10^9^	2 mL kg^−1^ seeds
		*Azospirillum brasilense* AbV6		
	*Bradyrhizobium* + *Azospirillum* (1:1)	*Bradyrhizobium japonicum* SEMIA 5079		2 mL kg^−1^ seeds + 2 mL kg^−1^ seeds
		*Bradyrhizobium diazoefficiens* SEMIA 5080	2.0 × 10^10^ +	
		*Azospirillum brasilense* AbV5	2.0 × 10^9^	
		*Azospirillum brasilense* AbV6		
	*Bradyrhizobium* + *Azospirillum* (2:1)	*Bradyrhizobium japonicum* SEMIA 5079		4 mL kg^−1^ seeds + 2 mL kg^−1^ seeds
		*Bradyrhizobium diazoefficiens* SEMIA 5080	2.0 × 10^10^ + 2.0 × 10^9^	
		*Azospirillum brasilense* AbV5		
		*Azospirillum brasilense* AbV6		

Basal fertilization consisted of 240 kg ha^−1^ of NPK 00-20-20 in Ponta Grossa and Bandeirantes and 300 kg ha^−1^ of NPK 00-20-20 in Ouro and Artur Nogueira. CFU: colony-forming unit; NPK: nitrogen–phosphorus–potassium fertilizer.

**Table 4:** Description of the microorganisms and nitrogen fertilization levels used in maize and wheat trials conducted in different regions during the 2023/2024 growing season.

Crop	Treatments	Microorganisms	Dose
Maize and wheat	Control	-	-
	100% Nitrogen fertilization (N)	-	120 kg N ha^−1^
	50% Nitrogen fertilization (N)	-	60 kg N ha^−1^
	75% Nitrogen fertilization (N)	-	90 kg N ha^−1^
	Commercial product	*Azospirillum brasilense* AbV5	2 mL kg^−1^ seeds
		*Azospirillum brasilense* AbV6	
	*Azospirillum brasilense*	*Azospirillum brasilense* AbV5	2 mL kg^−1^ seeds
		*Azospirillum brasilense* AbV6	
	*Azospirillum* + 50% N	*Azospirillum brasilense* AbV5	60 kg N ha^−1^
		*Azospirillum brasilense* AbV6	2 mL kg^−1^ seeds
	*Azospirillum* + 75% N	*Azospirillum brasilense* AbV5	90 kg N ha^−1^
		*Azospirillum brasilense* AbV6	2 mL kg^−1^ seeds
	*Azospirillum* + 100% N	*Azospirillum brasilense* AbV5	120 kg N ha^−1^
		*Azospirillum brasilense* AbV6	2 mL kg^−1^ seeds

For maize, basal fertilization consisted of 300 kg ha^−1^ of NPK 00-20-20 in Ponta Grossa and Ouro, 240 kg ha^−1^ of NPK 00-20-20 in Bandeirantes, and 300 kg ha^−1^ of NPK 00-21-21 in Artur Nogueira. For wheat, basal fertilization consisted of 300 kg ha^−1^ of NPK 10-20-10 at all four locations. CFU: colony-forming unit; N: nitrogen; NPK: nitrogen–phosphorus–potassium fertilizer.

**Table 5:** Joint analysis of soybean crop production components under nitrogen fertilization and inoculation/co-inoculation with *Bradyrhizobium* spp. and *Azospirillum brasilense* at different doses during the 2023/2024 growing season in different regions.

Treatments	Production Components		
	No. of Pods Plant^−1^	TSW (g)	Productivity (kg ha^−1^)
Control	45.24	165.08	3545.88
Nitrogen fertilization	55.27 *	168.86	3737.31 *
*Bradyrhizobium*	54.28 *	175.50 *	3770.81 *
*Azospirillum*	48.79	171.89	3756.83 *
*Bradyrhizobium* + *Azospirillum* (1:1)	47.91	178.82 *	3628.02
*Bradyrhizobium* + *Azospirillum* (2:1)	52.43 *	179.06 *	3810.65 *
**Average**	50.65	173.20	3708.25
**C.V. (%)**	10.34	4.36	3.34

* indicates a statistical difference from the control by Dunnett’s test at a 5% probability. TSW—thousand-seed weight; C.V.—coefficient of variation.

**Table 6:** Joint analysis of shoot and root dry masses in the V4, flowering and R3 stages of maize inoculated with *Azospirillum brasilense* and/or with nitrogen fertilization doses in the 2023/2024 harvest in different regions.

Treatments	Dry Mass (g)											
	Aerial Part						Roots					
	V4		Flowering		R3		V4		Flowering		R3	
Control	237.19		2347.50		2470.63		65.13		551.38		587.69	
100% nitrogen fertilization (N)	274.13	*	2580.63	*	2558.13		69.31		601.25		648.69	*
50% nitrogen fertilization (N)	249.88		2456.88		2528.75		68.12		579.44		633.50	
Commercial product	269.50	*	2579.38	*	2627.81	*	69.35		589.63		632.38	
*Azospirillum brasilense*	273.75	*	2532.50	*	2570.83		69.90		560.92		639.75	*
*Azospirillum* + 50% N	268.25	*	2591.25	*	2575.63		67.23		601.56		642.63	*
*Azospirillum* + 75% N	269.63	*	2705.00	*	2585.00		68.58		620.44	*	646.94	*
*Azospirillum* + 100% N	274.81	*	2708.13	*	2589.38		70.71	*	633.81	*	642.63	*
**Average**	264.64		2562.66		2563.27		68.54		592.30		634.27	
**C.V. (%)**	7.93		4.27		4.26		5.93		8.71		6.74	

* indicates a statistical difference by Dunnett’s test at a 5% probability of error. N—nitrogen; C.V.—coefficient of variation.

**Table 7:** Joint analysis of the production components of the maize crop inoculated with *Azospirillum brasilense* and/or with nitrogen fertilization doses in the 2023/2024 harvest in different regions.

Treatments	(Production Components)						
	Number of Rows of Grains per Ear^−1^	No. of Grains per Row^−1^		TSW (g)		Productivity (kg ha^−1^)	
Control	15.56	34.73		266.02		8431.63	
100% nitrogen fertilization (N)	16.19	39.50	*	277.88		8833.28	*
50% nitrogen fertilization (N)	15.94	37.21		280.44		8650.89	
Commercial product	16.44	36.54		281.82		8715.74	
*Azospirillum brasilense*	16.08	37.62		271.42		8703.36	
*Azospirillum* + 50% N	16.19	36.75		279.92		8895.47	*
*Azospirillum* + 75% N	16.31	38.36		285.48	*	9033.72	*
*Azospirillum* + 100% N	16.75	38.64	*	283.75		9114.53	*
**Average**	16.18	37.42		278.34		8797.33	
**C.V. (%)**	6.71	7.86		5.49		3.65	

* indicates a statistical difference by Dunnett’s test at a 5% probability of error. N—nitrogen; C.V.—coefficient of variation.

**Table 8:** Joint analysis of shoot and root dry masses at the tillering, flowering and maturation stages of wheat inoculated with *Azospirillum brasilense* and/or with nitrogen fertilization doses in the 2023/2024 harvest in different regions.

Treatments	Dry Mass (g)											
	Aerial Part						Roots					
	Tillering		Flowering		Maturation		Tillering		Flowering		Maturation	
Control	41.18		160.08		205.33		39.77		111.32		109.12	
100% nitrogen fertilization (N)	44.98		188.75	*	229.93	*	43.72		127.46	*	123.34	*
50% nitrogen fertilization (N)	44.50		168.88		214.03		42.94		122.93		114.87	
Commercial product	47.93		171.20		218.01		42.60		128.57	*	114.75	
*Azospirillum brasilense*	50.83	*	188.96	*	197.71		46.17		129.17	*	110.92	
*Azospirillum* + 50% N	46.97		178.77	*	218.14		45.63		131.91	*	115.31	
*Azospirillum* + 75% N	53.07	*	187.77	*	228.15	*	49.07	*	130.02	*	122.96	*
*Azospirillum* + 100% N	49.25		195.66	*	235.97	*	50.56	*	136.32	*	125.13	*
**Average**	47.34		180.01		218.41		45.06		127.21		117.05	
**C.V. (%)**	16.08		8.57		6.53		12.81		8.74		7.02	

* indicates a statistical difference by Dunnett’s test at a 5% probability of error. N—nitrogen; C.V.—coefficient of variation.

**Table 9:** Joint analysis of production components of wheat inoculated with *Azospirillum brasilense* and/or subjected to different nitrogen fertilization levels during the 2023/2024 growing season across different regions.

Treatments	(Production Components)				
	No. of Plants m^2−1^	No. of Ears m^2−1^	No. of Spikelets ear^−1^	No. of Grains ear^−1^	Productivity (kg ha^−1^)
Control	230.44	319.38	26.53	24.84	3215.63
100% nitrogen fertilization (N)	235.81	337.31 *	29.65 *	28.95 *	3592.60 *
50% nitrogen fertilization (N)	235.81	331.63	28.42	26.98	3454.66 *
Commercial product	238.75	334.06	28.68	26.97	3423.77 *
*Azospirillum brasilense*	250.50 *	286.17	28.37	26.69	3552.53 *
*Azospirillum* + 50% N	235.75	343.69 *	30.59 *	27.83 *	3554.63 *
*Azospirillum* + 75% N	237.56	352.13 *	30.40 *	29.17 *	3614.78 *
*Azospirillum* + 100% N	240.06	353.73 *	32.38 *	29.65 *	3681.12 *
**Average**	238.09	332.26	29.38	27.64	3511.21
**C.V. (%)**	3.84	4.25	5.23	5.57	3.59

* indicates a statistical difference by Dunnett’s test at a 5% probability of error. N—nitrogen; C.V.—coefficient of variation.

## Data Availability

The data supporting the findings of this study are available from the corresponding author upon reasonable request.
